# 1102. Case Study. Use of Bromelain-based Enzymatic Debridement with Skin Cell Suspension Autograft Application for Deep Facial Burns

**DOI:** 10.1093/jbcr/irag033.206

**Published:** 2026-04-06

**Authors:** Aaron Hong, Manasa Gujju, Jonathan E Schoen, Jeffrey E Carter, M Victoria P Miles

**Affiliations:** University Medical Center Burn Center, Louisiana State University Health Sciences Center, New Orleans, New Orleans, LA; Tulane School of Medicine, New Orleans, LA; UCI Health Regional Burn Center, New Orleans, LA; University Medical Center Burn Center, Louisiana State University Health Sciences Center New Orleans, New Orleans, New Orleans, LA; University Medical Center Burn Center, Louisiana State University Health Sciences Center, Department of Surgery, New Orleans, New Orleans, LA

## Abstract

**Patient Presentation (age range, injury details, relevant history):**

An ABA-verified center treated five patients between 12/2024 and 8/2025 with 16-67% total body surface area (TBSA) thermal burns (including the face) with Bromelain-based enzymatic debridement (BBED) followed by skin cell suspension autograft (SCSA) application to the face at the time of their initial burn excision. For each patient, the timing, technique, and outcomes of facial debridement were recorded, with particular focus on wound healing, complications, and hospital length of stay (LOS).

**Clinical Challenges:**

The goal of acute burn excision is timely and effective removal of irreversibly injured tissue while preserving viable dermis. Traditional surgical debridement techniques are efficacious in removing nonviable tissue, but they are also non-selective and may result in unnecessary tissue loss, bleeding, and delayed wound closure. This is especially relevant for deep burns of the face, as they carry great risk of functional and cosmetic impairment from scarring. Bromelain-based enzymatic debridement (BBED) was developed as a debridement alternative, utilizing enzymes derived from pineapple stems to selectively remove necrotic tissue while sparing healthy dermis. However, the use of BBED for deep facial burns remains underreported. This case series details the use of BBED with immediate skin cell suspension autograft (SCSA) application for facial burns in five patients, highlighting procedural details, outcomes, and comparison with the current standard of care.

**Management Approach:**

Patients underwent debridement of their facial burns with BBED followed by immediate SCSA application at the time of their index burn excision (post-burn day 3-5). The enzyme was applied to the entire burned facial area for 65-150 minutes, resulting in excellent eschar removal and preservation of viable tissue.

**Outcomes:**

For the first two patients, SCSA was reapplied at the subsequent operation; this was abandoned in the later cases as it was deemed unnecessary for healing. No complications related to the enzymatic procedure were observed. All patients achieved rapid wound healing, excellent repigmentation, favorable cosmetic outcomes, and demonstrated a LOS less than the BCQP national average (2-4 days/%TBSA) at an average 0.91 days/%TBSA.

**Lessons Learned:**

This case series showcases the use of BBED followed by immediate SCSA application as an effective modality for managing deep facial burns in patients with large TBSA burn injuries. These findings support a role of enzymatic debridement with SCSA in the management of deep facial burns, offering benefits for both functional and cosmetic recovery.

**Applicability to Practice:**

This case series showcases the feasibility of using BBED with SCSA application for the treatment of deep facial burns while maximizing healing, cosmetic, and functional outcomes.

